# ZBTB7A functioned as an oncogene in colorectal cancer

**DOI:** 10.1186/s12876-020-01456-z

**Published:** 2020-11-09

**Authors:** Li Wang, Meng-Xia Zhang, Mei-Fang Zhang, Zi-Wei Tu

**Affiliations:** 1 Department of Radiotherapy, Eye & ENT Hospital, Shanghai Medical College, Fudan University, Shanghai, 200000 China; 2 Department of Nasopharyngeal Carcinoma, State Key Laboratory of Oncology in South China, Collaborative Innovation Center for Cancer Medicine, Sun Yat-sen University Cancer Center, Guangzhou, 510060 Guangdong China; 3 Department of Pathology, Sun Yat-sen University Cancer Center, State Key Laboratory of Oncology in South China, Collaborative Innovation Center for Cancer Medicine, Guangzhou, 510060 Guangdong China; 4 Department of Radiotherapy, Jiangxi Cancer Hospital, Medical College, Nanchang University, No. 519, Beijing East Road, Qingshan Lake District, Nanchang, 330029 Jiangxi China

**Keywords:** ZBTB7A, Oncogene, Colorectal cancer (CRC), Prognosis, Biomarker

## Abstract

**Background:**

Despite zinc finger and BTB domain-containing 7A (ZBTB7A) documented importance in multiple tumors, the function and clinical value in Colorectal cancer (CRC) remain elusive. The aim of this study was to evaluate the functional roles and the clinical value of ZBTB7A in CRC progression.

**Methods:**

The level of ZBTB7A was detected in a large cohort of CRC patients (*n* = 189) by immunohistochemistry (IHC), and we analyzed the diagnostic and prognostic value of the protein. In addition, the functional roles of ZBTB7A on CRC were explored in vitro and in vivo.

**Results:**

Survival analyses indicated that patients with high ZBTB7A expression made the prognosis worse (*P* = 0.024). Functionally, knockdown of ZBTB7A could markedly inhibit tumor proliferation in vitro and in vivo, whereas ZBTB7A overexpression displayed the opposite results.

**Conclusions:**

ZBTB7A was associated with poor survival outcomes and functioned as an oncogene in CRC patients, indicating that it is a potential prognostic biomarker and therapeutic target for CRC patients.

## Background

With nearly 1.8 million new cases and 900,000 deaths annually, colorectal cancer (CRC) is the third most popular malignant tumor worldwide [1, 2]. Like other cancers, CRC is induced by a multistep genetic disorder and involves accumulation of both genetic and epigenetic changes [3, 4]. Nowadays, the major treatment methods for CRC include surgical resection, radiotherapy, chemotherapy, targeted molecular therapy, and immunotherapy, which have a great development over the last few decades [5, 6]. However, the recurrence and metastasis occur in numerous advanced patients who undergo systemic therapy and the survival rates for advanced patients remains low. Still, our knowledge on CRC is limited [7, 8]. Thus, the further identification of the molecular mechanisms of CRC carcinogenesis and more effective treatment approaches are urgently required to increase early detection and reduce mortality from CRC.

Zinc finger and BTB domain-containing 7A (ZBTB7A, also known as FBI, POKEMON and LRF) is a member of the POZ/BTB and Krüppel (POK) family of transcriptional repressors [9, 10]. The gene was firstly identified by Davies JM et al. in 1999 and encodes a 62 kDa Zn-finger protein [11]. Initially, ZBTB7A is reported as an oncoprotein for directly inhibiting the expression of tumor suppressor gene ARF in non-Hodgkin lymphoma tissues [9]. Recently, overexpression of ZBTB7A has been found in multiple tumors, including lung cancer [12, 13], breast cancer [14], oral carcinoma [15], and ovarian cancer [16]. In addition, some opposite functions of ZBTB7A have been reported in other kinds of cancers, such as melanoma and prostate cancer [17, 18]. It follows that ZBTB7A plays a context-dependent role on tumorigenesis and progression. As for CRC, Joo et al. reported that ZBTB7A was up-regulated in CRC cell lines and tissues [19]. Zhao et al. found that silencing ZBTB7A attenuated the proliferation of CRC cell lines SW480 and SW620 [20]. Zhu et al. revealed that overexpression of ZBTB7A markedly promoted the growth of CRC cells including LoVo, HR8348, and HT29; while silencing ZBTB7A inhibited the growth of LoVo, HR8348, and HT29 cells [21]. These studies suggested that ZBTB7A would enhance the proliferation ability of CRC cells. However, these literatures did not comprehensively describe the role of ZBTB6A on proliferation of CRC through in vivo and in vitro assays, and the prognostic values of ZBTB7A were not fully studied.

In the current study, we aimed to verify and further uncover the functional role and clinical significance of ZBTB7A in CRC using two distinct CRC cell lines HCT116 and DLD1 and a relatively larger cohort of patients (*n* = 189). The results of this study may provide an important biomarker for clinical diagnosis and treatment of CRC.

## Methods

### Patient tissue specimens

For tissue samples, a total of 189 paraffin-embedded primary specimens of CRC patients were collected in our study. All CRC patients underwent surgical resection from 2000 to 2007 at the Sun Yat-Sen University Cancer Center (SYSUCC). No patient received preoperative radiotherapy or chemotherapy. Written informed consent was obtained from the sample donors, and approval was granted by the Institute Research Medical Ethics Committee of Sun Yat-Sen University.

### Cell lines and cell culture

For cells, the human CRC cell lines HCT116 and DLD1 were obtained from Cell Bank of Chinese Academy of Sciences (Shanghai, China). HCT116 and DLD1 stable cell lines were cultured at 37 °C in DMEM medium (Invitrogen) with 10% fetal bovine serum (Gibco, USA) in a humidified incubator with 5% CO_2_.

### Plasmids and antibodies

The full-length cDNAs of human ZBTB7A was cloned into the pSin-EF2-puro vector to generate the stable overexpression of ZBTB7A in HCT116 cells. Two RNA interference lentiviral vectors (shRNA-ZBTB7A 1 and 2) for silencing ZBTB7A in DLD1 cells were constructed and synthesized by Shanghai Genechem Technology Co., Ltd. Human anti-ZBTB7A (ABGENT, USA) and the GAPDH (Abcam, UK) antibodies were used. The targeted sequences of ZBTB7A were as follows: (shRNA-1) 5′-GCAGAAGGTGGAGAAGAAGAT-3′ and (shRNA-2) 5′-CCAGTACTTCAAGAAGCTGTT-3′. The sequence of shRNA-NC was 5′-ccgcag gtatgcacgcgt-3′.

### RNA extraction and qRT-PCR

The total RNA from each group was extracted with RNA simple Total RNA Kit (TIANGEN, China). Then the cDNA was synthesized by the PrimeScript™ RT Reagent Kit (Takara, Japan). And the qPCR was performed to detect the expression level of ZBTB7A mRNA with TB Green™ Premix Ex Taq™ II (Takara, Japan). The primer sequences involved in our study were as follows:

- For ZBTB7A:5′-TTGCCAAAGATACCTGCTGA-3′(forward),
- 5′-ACAGTCAGCCGCATCTTCTT-3′(reverse);
- for GAPDH:5′-ACAGTCAGCCGCATCTTCTT-3′ (forward),
- 5′-GACAAGCTTCCCGTTCTCAG-3′ (reverse).

### Western blot assay

The total protein was extracted from CRC cells by RIPA Lysis Buffer (Beyotime, China). A total of 30 μg harvested protein was loaded, subjected to 10% SDS-PAGE, and then transferred to PVDF membranes. The membranes were blocked with 5% non-fat milk for 1 h on shaking table. Next, membranes were incubated with primary antibody (ZBTB7A and GAPDH) at 4 ∘C overnight and secondary antibody for 1 h the next day. Finally, the membranes were detected by the ECL chemiluminescence system (Pierce, Rockford, USA).

### Plate colony formation assay

Briefly, 600 cells (DLD1-knock-down, HCT116-overexpression and their control cells) were seed into 6-well plates and cultured for 14 days. The cells were incubated at 37 ∘C for about 14 days. Then the colonies were fixed with 100% methyl alcoholand for 10 min then stained with crystal violet for 20 min, at room temperature. Cell colonies were calculated and photographed.

### CCK8 assay

The cell viability in vitro was assessed using The CCK8 assay was used to assess the CRC cell viability. In brief, cells (1000/well) were seeded in 96-well plates and incubated for consecutive 5 days. Ten microliters of CCK8 solution (Cell Counting Kit-8, Beyotime, China) was added to each well and incubated for 1.5 h. The absorbance value (OD) of each well was measured spectrophotometrically at 450 nM by automatic microplate reader.

### Tumor xenograft formation assay in vivo

All the experiments were approved by the Ethical Committee of the Sun Yat-Sen University Cancer Center. The five-week-old male athymic nude mice were purchased from Shanghai Institutes for Biological Sciences (Shanghai, China). The mice were randomly divided into four groups (*n* = 6 for each group). ZBTB7A-knockdown and control DLD1 cells (5 × 10^6^/0.2 ml PBS), ZBTB7A-overexpression and control HCT116 cells (5 × 10^6^/0.2 ml PBS) were injected into the flanks of mice, respectively. Tumor size was measured once every 2 days with digital calipers. The tumor volume was calculated as 1/2(length×width^2^). After about 20 days, mice were sacrificed. Then the tumor tissues were preserved for further hematoxylin and eosin (H&E) and IHC staining.

### Immunohistochemistry (IHC)

The expression level of ZBTB7A in CRC specimens was measured by IHC. Briefly, the sections were sequentially deparaffinized by dimethylbenzene, rehydrated by graded ethanol, repaired for antigen by citrate buffer (pH 6.0), and incubated with primary anti-ZBTB7A antibody diluted 1:500 at 4 ∘C overnight in a humidified container. After three washes with 1× PBS, sections were incubated with the secondary antibody for 1 h at room temperature and then immunostained with 3, 3′-diaminobenzidine tetrahydrochloride (DAB) chromagen kit. The IHC results were calculated by multiplying the intensity degrees and positive rates. The scores were categorized into 0–3 (no staining, weak staining, moderate staining and high staining) for staining intensity and 0–4 (no staining, < 10, 10–50%, 50–80%, and > 80%) for the positive ratio. The final scores (0 to 12) were grouped into no/low expression (≤1) and high expression (> 1). The scores were calculated blindly by two pathologists.

### Statistical analysis

Statistically significant differences were investigated by SPSS software (version 19.0). T-test was used to compare the differences among continuous parameters. Chi-square test or Fisher exact test was used to compare clinicopathological variables between different ZBTB7A expression groups. The endpoint of overall survival (OS) was measured from the first date of treatment to the date of death due to any causes. Survival analysis was assessed by the Kaplan-Meier method and the log-rank test. *P* value less than 0.05 was considered statistically significant. The graphs were generated by GraphPad Prism 5.0. The raw data of this paper have been uploaded onto the Research Data Deposit (RDD) with an RDD number of RDDB2020000904.

## Results

### High expression of ZBTB7A is correlated with poor outcomes in CRC patients

**Fig. 1:**
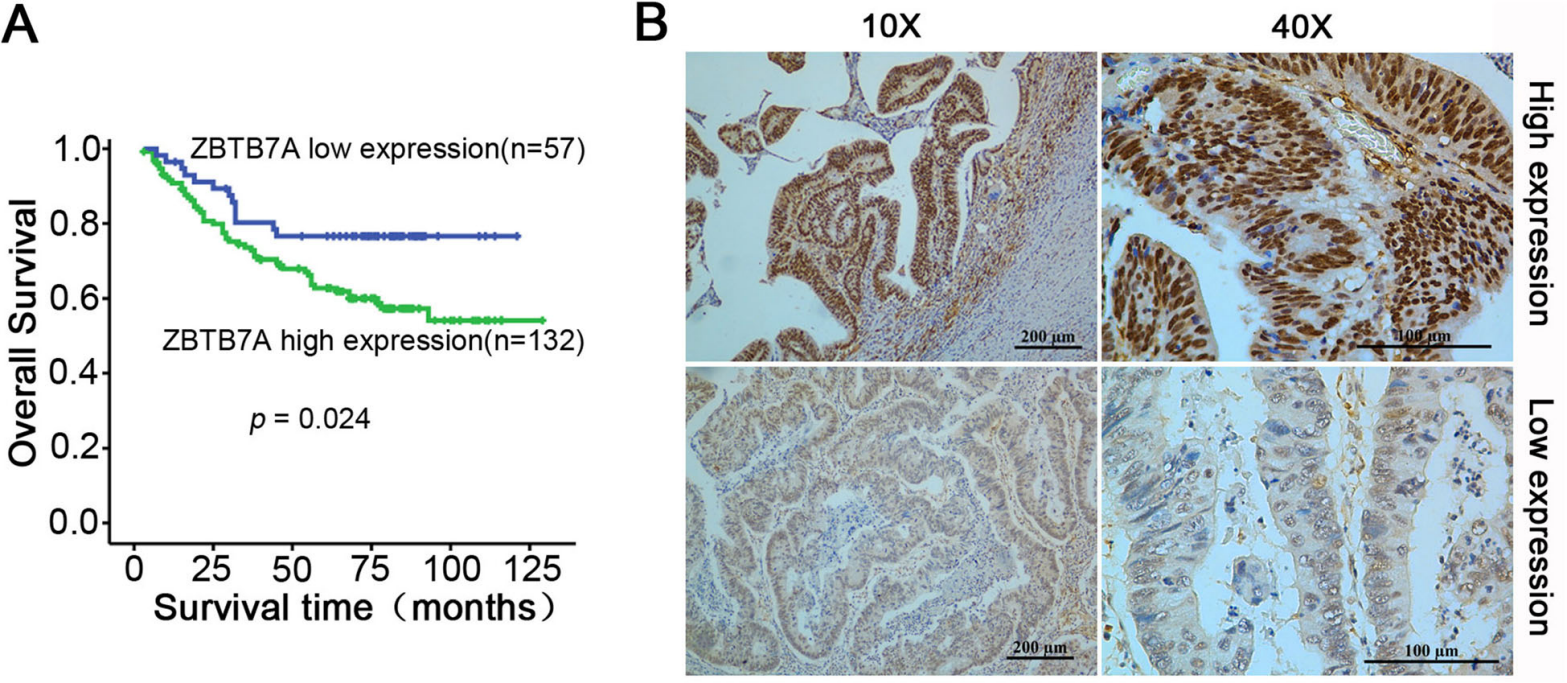
ZBTB7A expression correlates with poor outcome in CRC patients. **a** Kaplan-Meier survival analysis was used to detect the clinical significance of ZBTB7A in overall survival (OS) of CRC patients. Patients with high expression of ZBTB7A presented lower OS compared to that of low expression of ZBTB7A. **b** The expression of ZBTB7A in CRC tissue was detected by IHC. The representative images of high and low expression of ZBTB7A were shown

We performed IHC staining for ZBTB7A in 189 paraffin-embedded CRC tissue samples and then analysed the clinical characteristics of high and low ZBTB7A expression groups. As shown in supplementary Table 1, patients with high expression of ZBTB7A had prominently higher rates of mortality (*p* = 0.033) and metastasis (*p* = 0.024). In addition, we further determined whether ZBTB7A was influential in the prognosis of patients by correlation analysis. As expected, the Kaplan-Meier analysis showed those CRC patients with high ZBTB7A expression had a markedly decreased overall survival (OS) rate compared with low ZBTB7A expression patients (Fig. 1a, *p* = 0.024), indicating that high ZBTB7A expression may have a trend toward poor prognosis in CRC patients.

### Knockdown of ZBTB7A attenuated cell proliferation in CRC

**Fig. 2:**
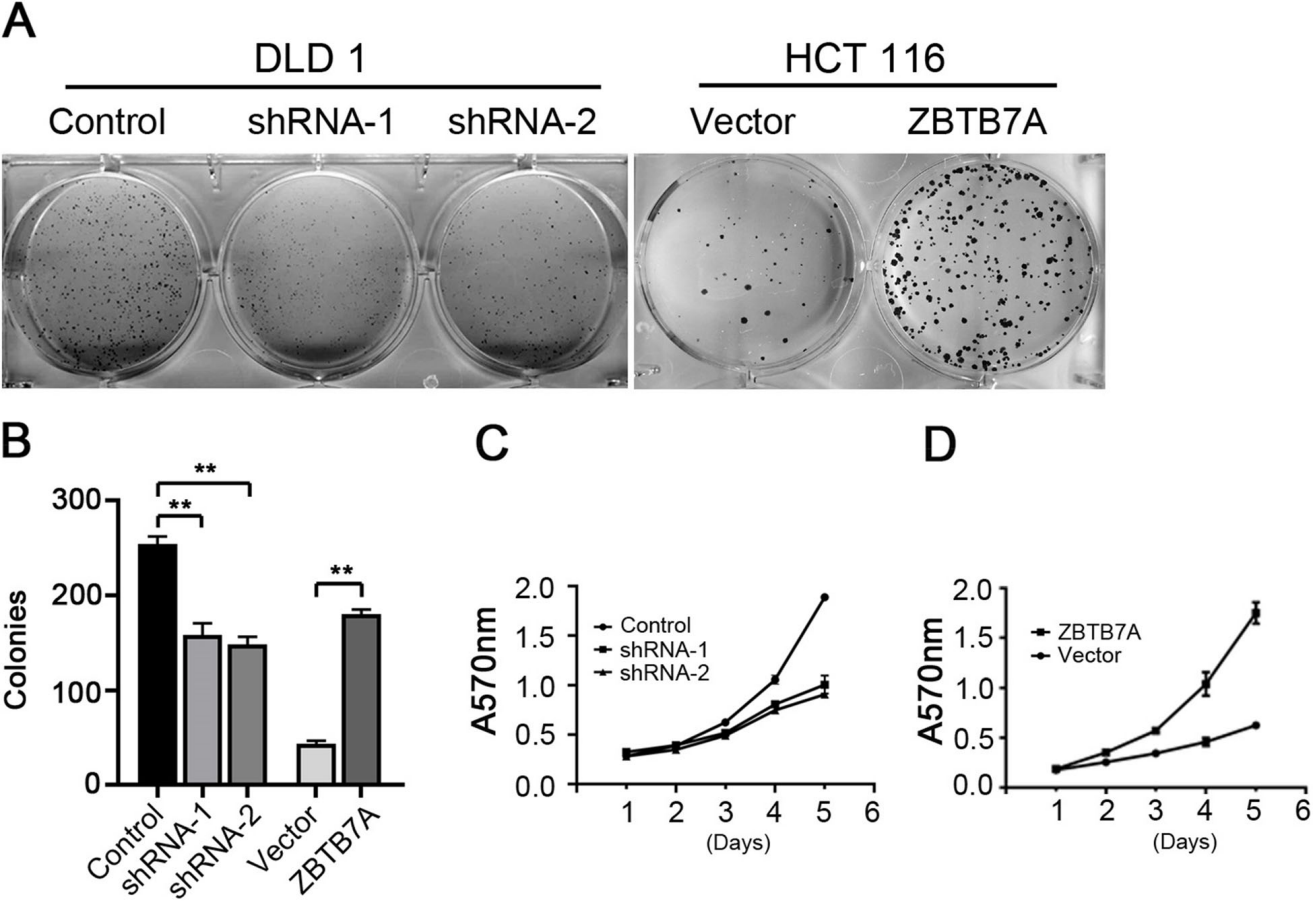
The protein and mRNA expression of ZBTB7A in the stable cell lines. **a** ZBTB7A expression in the stable cell lines was confirmed at the protein level by Western blot. **b**, **c** The expression of ZBTB7A in the stable cell lines was confirmed at the mRNA level by qPCR (** *p* < 0.01)

**Fig. 3:**
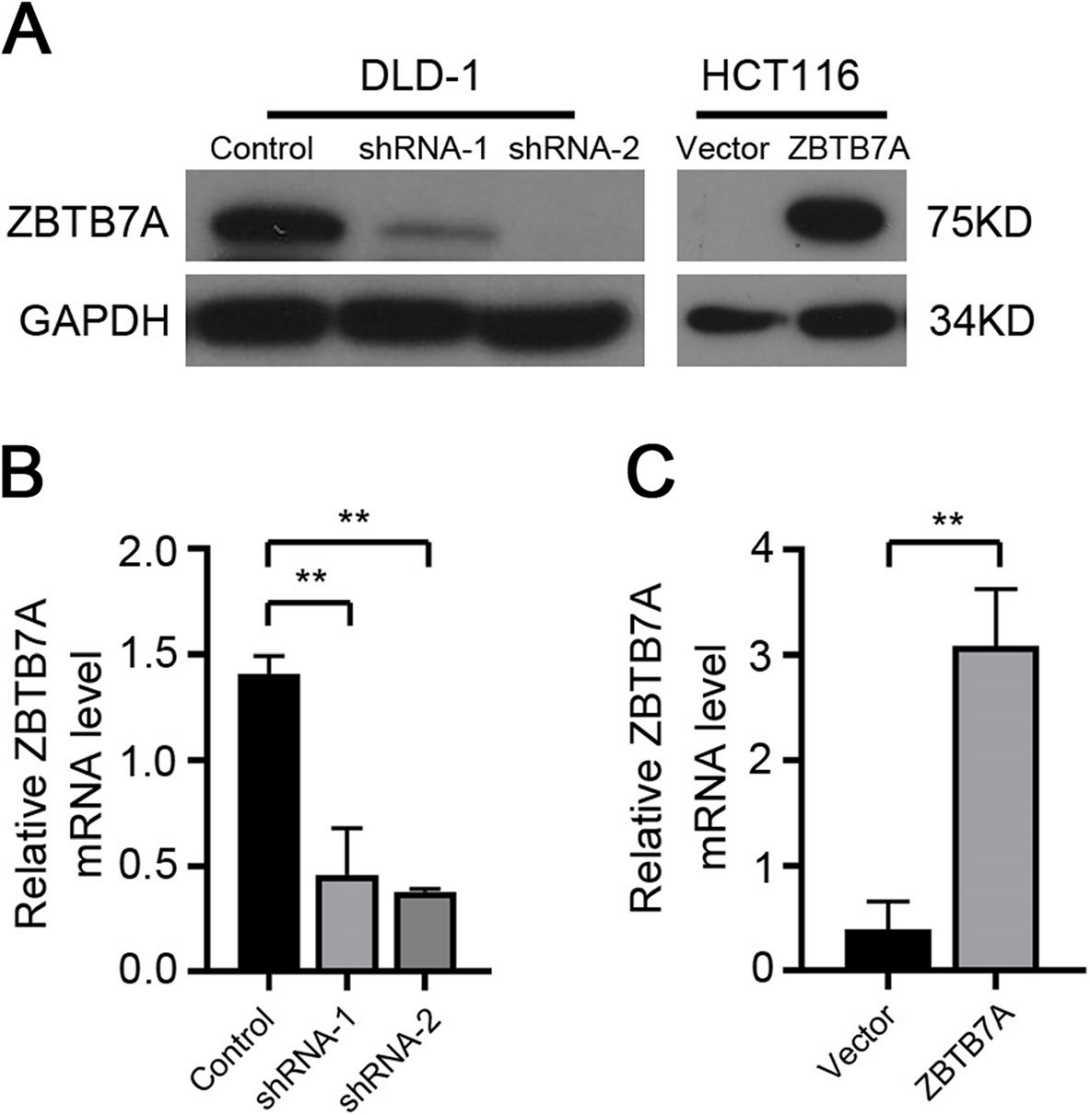
ZBTB7A promotes cell proliferation of CRC cells in vitro. **a, b** Cell growth capacity was evaluated by colony formation for about 2 weeks. Silencing ZBTB7A significantly inhibited the growth of DLD1 cells, while upregulation of ZBTB7A evidently promoted the growth of HCT116 cells. **c, d** Cell viability was analyzed using CCK8 assays in CRC cells. Cell proliferation of knock-down cells and overexpression cells were attenuated and enhanced, respectively

As its potential pro-tumor function of ZBTB7A in CRC, the effect of ZBTB7A in cell proliferation was further investigated in vitro. Two different shRNAs specifically targeting different ZBTB7A coding regions (shRNA-1 and shRNA-2) were stably expressed in DLD1 cell line, and we generated the stable overexpression of ZBTB7A in HCT116 cells (Fig. 2). Colony formation assay indicated that knockdown of ZBTB7A inhibited the ability of colony formation of DLD1 cells, whereas overexpression of ZBTB7A resulted in increased colonies in HCT116 cells (Fig. 3a, b). The effect of ZBTB7A on cell proliferation was assessed by CCK8 method. The results showed that cell viability was significantly inhibited when ZBTB7A was knocked down in DLD1 cells (Fig. 3c). On the contrary, stably overexpression of ZBTB7A in HCT116 cells markedly enhanced cell proliferation (Fig. 3d). Altogether, these results indicate that ZBTB7A promoted cell proliferation in CRC cells.

### ZBTB7A promotes tumorigenicity in CRC cells in vivo

**Fig. 4:**
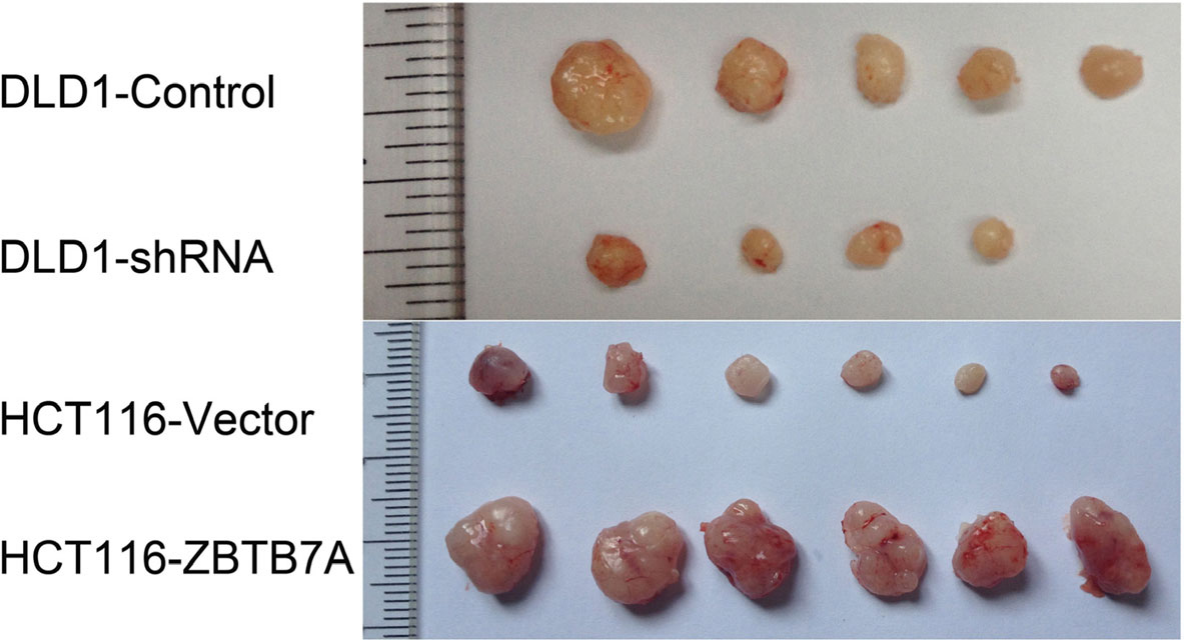
Overexpression of ZBTB7A promotes tumor growth of CRC cells in vivo. The number of 5 × 10^6^ ZBTB7A-overexpressing or ZBTB7A-silencing or their control cells were subcutaneously injected into male athymic nude mice for tumor growth measurement. Representative tumor images of each group are shown

To further explore the effect of ZBTB7A on tumor growth, we detected the tumorigenesis ability through the xenograft tumor model in vivo over 2 weeks. As assessed by tumor volume, knockdown of ZBTB7A significantly inhibited CRC growth, while ectopic ZBTB7A expression markedly promoted CRC cell tumorigenesis in vivo (Fig. 4). The above results revealed that ZBTB7A can promote the proliferation of CRC in vivo.

## Discussion

ZBTB7A has been proved to be a critical factor for diagnosis, prognostication and prediction of treatment effect in multiple types of cancer [12–16]. Although a few studies have identified ZBTB7A as a functional gene in CRC, the functional studies were not comprehensive [19–22]. In the current study, we re-identified the functional role of ZBTB7A in two distinct CRC cell lines (DLD1 and HCT116) through in vitro and in vivo assays. Furthermore, we detected the association of ZB7B7A with survival outcomes of CRC patients based on a relatively large cohort (*n* = 189). Our results demonstrated that ZBTB7A was related to the proliferation of CRC cells and was a poor prognostic factor for CRC patients.

Several studies have investigated the expression of ZBTB7A in CRC. Joo et al. reported that the expression of ZBTB7A was significantly higher in CRC cells and tissues than those in high-grade dysplasia or normal mucosa [19]. Zhao and colleagues demonstrated that ZBTB7A mRNA and protein was up-regulated in CRC tissue when compared to that in adjacent normal tissues [20]. However, these studies only detected the expression discrepancies between tumor tissues and dysplasia diseases or normal mucosa, the association between ZBTB7A and prognosis of CRC patients remains unknown. In our study, the IHC staining results demonstrated that ZBTB7A was mainly localized in the cytoblast**,** and high expression of ZBTB7A was markedly correlated with mortality and metastasis, but not with TNM stage or Ducks’ Stage in CRC patients. On the contrary, higher expression of ZBTB7A was associated with lymph node metastasis and higher Duke’s stage in a previous study [22]. The discrepancy may be due to the larger cohort used in our study. Furthermore, patients with high ZBTB7A expression had a shorter overall survival (OS) compared to that of low ZBTB7A expression group. These results indicated that ZBTB7A may serve as a promising biomarker for prognosis of CRC patients.

ZBTB7A have shown to play a critical role in the tumorigenesis [22, 23]. Zhu and colleagues reported that ZBTB7A accelerated cellular proliferation and invasion of CRC cells through enhancing E26 transformation specific proto-oncogene 1 signaling activity [21]. Zhao et al. reported that silencing ZBTB7A significantly decreased the growth of CRC cells (SW480 and SW620) in vitro [20]. Yet, the role of ZBTB7A on CRC proliferation is investigated in vitro and in vivo studies are missing. Thus, we re-identified the effect of ZBTB7A on CRC proliferation both in vitro and in vivo. Our study comfirmed that knockdown of ZBTB7A inhibited the proliferation of DLD1 cells in vitro and repress the tumorigenesis of DLD1 cells in vivo. Conversely, up-regulation of ZBTB7A promoted the growth of HCT116 cells both in vitro and in vivo. The results suggest that ZBTB7A functions as an oncogene in CRC.

Mechanically, ZBTB7A could act as a tumor suppressor by directly binding to the ERα promoter in ERα-positive breast tumors [24]. Han et al. found that ZBTB7A acted as a regulatory factor by binding to genomic promoters and enhancers with the help of NF-kappa B and multiple other transcription factors [25]. Zhang et al. reported that ZBTB7A could enhance Osteosarcoma chemoresistance by transcriptionally repressing lncRNALINC00473-IL24 activity [26]. As for CRC, Zhu and colleagues presented a link between ZBTB7A and the decrease of p53 expression in CRC and the abnormality of p53 contributes to CRC cell survival through regulating apoptosis [27]. Zhao reported that ZBTB7A might play a key role in the proliferation, progression and apoptosis of CRC independently of p14^ARF^–MDM2–p53 pathway [22]. Whereas few studies concerning the mechanisms of ZBTB7A on CRC have been reported, further experiments to reveal the molecular mechanism of ZBTB7A on the carcinogenesis and survival of CRC are needed.

### Limitations

Although the results were significant, there are still some limitations in our study. Firstly, the molecular mechanism of ZBTB7A in CRC has not been investigated. Sencondly, all the tissue samples were from the same cancer center. Thirdly, although we found ZBTB7A expression was significantly associated with distant metastasis, we didn’t introduce ZBTB7A knockdown or overexpression system into the migration or invasion functional experiments. Future study is still needed to further identify the function of ZBTB7A and underly the molecular mechanisms in CRC.

## Conclusions

To summarize, this study showed that high ZBTB7A expression was prominently related with shorter overall survival in CRC patients, and could promote the proliferation ability of CRC cells in vitro and induce tumorigenesis in vivo. Our results suggest that ZBTB7A is a potential biomarker for clinical diagnosis and treatment of CRC patients.

## Supplementary information

**Additional file 1: Supplementary Table 1**. Clinicopathological characteristics of high or low ZBTB7A expression groups in patients with CRC.

**Additional file 2.**

## Data Availability

The datasets generated and/or analysed during the current study are available in the Research Data Deposit (RDD) with an RDD number of RDDB2020000904.

## Abbreviations

CRC: Colorectal cancer
ZBTB7A: Zinc finger and BTB domain-containing 7A
IHC: Immunohistochemistry
POK: POZ/BTB and Krüppel
SYSUCC: Sun Yat-Sen University Cancer Center
DAB: 3, 3′-diaminobenzidine tetrahydrochloride
OS: Overall survival
RDD: Research Data Deposit
