# Supplementary material for: ZBTB7A functioned as an oncogene in colorectal cancer
Source: BMC Gastroenterol. 2020 Nov 9;20:370. doi: 10.1186/s12876-020-01456-z (PMC7650168; doi:10.1186/s12876-020-01456-z)
Supplement: Supplementary file 1 — Additional file 1: Supplementary Table 1. Clinicopathological characteristics of high or low ZBTB7A expression groups in patients with CRC. [file 12876_2020_1456_MOESM1_ESM.docx]

**Supplementary table 1**

**Clinicopathological characteristics of high or low ZBTB7A expression groups in patients with CRC**

| Characteristics | Cases | ZBTB7A (%) | | P value |
| --- | --- | --- | --- | --- |
|  |  | Low expression | High expression |  |
| **Gender** |  |  |  | 0.560 |
| Male | 106 | 32 | 74 |  |
| Female | 83 | 25 | 58 |  |
| **Age** |  |  |  | 0.185 |
| <50 | 67 | 17 | 50 |  |
| ≥50 | 122 | 40 | 82 |  |
| **Clinical stage** |  |  |  | 0.134 |
| I-II | 83 | 29 | 54 |  |
| III-IV | 106 | 28 | 78 |  |
| **T stage** |  |  |  | 0.427 |
| T1-T2 | 40 | 13 | 27 |  |
| T3-T4 | 149 | 44 | 105 |  |
| **N stage** |  |  |  | 0.166 |
| N0 | 91 | 31 | 60 |  |
| N1-N3 | 98 | 26 | 72 |  |
| **Ducks’ stage** |  |  |  | 0.134 |
| A-B | 83 | 29 | 54 |  |
| C-D | 106 | 28 | 78 |  |
| **Death** |  |  |  | **0.033*** |
| Yes | 66 | 13 | 53 |  |
| No | 123 | 44 | 79 |  |
| **Relapse** |  |  |  | 0.476 |
| Yes | 5 | 2 | 3 |  |
| No | 184 | 55 | 129 |  |
| **Metastasis** |  |  |  | **0.024 *** |
| Yes | 53 | 10 | 43 |  |
| No | 136 | 47 | 89 |  |
