# Supplementary figures and images for: ZBTB7A functioned as an oncogene in colorectal cancer
Source: BMC Gastroenterol. 2020 Nov 9;20:370. doi: 10.1186/s12876-020-01456-z (PMC7650168; doi:10.1186/s12876-020-01456-z)

## Knock-down ZBTB7A

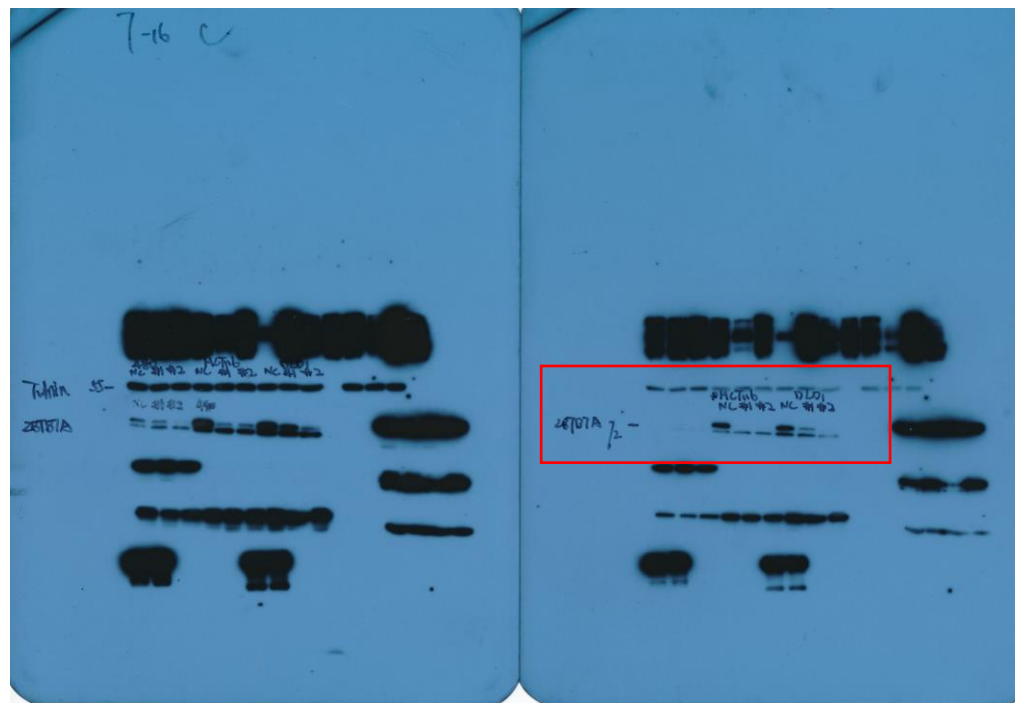

## Overexpression ZBTB7A

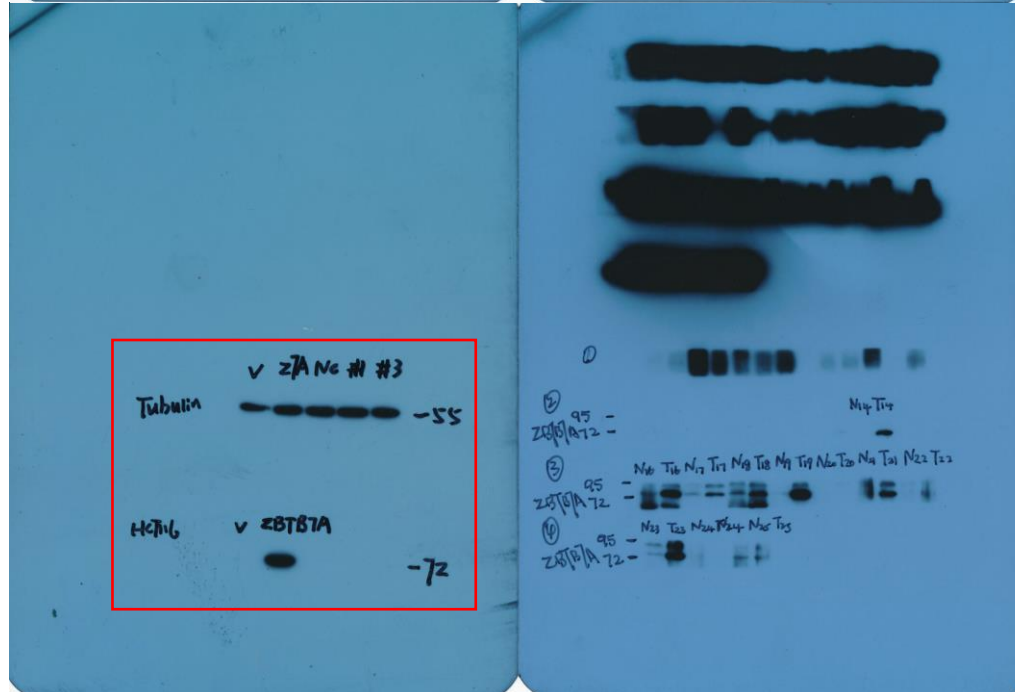

Supplement: Supplementary file 2 — Additional file 2. [file 12876_2020_1456_MOESM2_ESM.pdf]
